# Selected Diagnosed Chronic Conditions by Sexual Orientation: A National Study of US Adults, 2013

**DOI:** 10.5888/pcd12.150292

**Published:** 2015-11-05

**Authors:** Brian W. Ward, Sarah S. Joestl, Adena M. Galinsky, James M. Dahlhamer

**Affiliations:** Author Affiliations: Sarah S. Joestl, Adena M. Galinsky, James M. Dahlhamer, National Center for Health Statistics, Hyattsville, Maryland.

## Abstract

**Introduction:**

Research is needed on chronic health conditions among lesbian, gay, and bisexual populations. The objective of this study was to examine 10 diagnosed chronic conditions, and multiple (≥2) chronic conditions (MCC), by sexual orientation among US adults.

**Methods:**

The 2013 National Health Interview Survey was used to generate age-adjusted prevalence rates and adjusted odds ratios of diagnosed chronic conditions and MCC for civilian, noninstitutionalized US adults who identified as gay/lesbian, straight, or bisexual, and separately for men and women. Chronic conditions were selected for this study on the basis of previous research.

**Results:**

Hypertension and arthritis were the most prevalent conditions for all groups. Gay/lesbian adults had a 4.7 percentage-point higher prevalence of cancer than bisexual adults, and a 5.6 percentage-point higher prevalence of arthritis and a 2.9 percentage point higher prevalence of hepatitis than straight adults. The prevalence of chronic obstructive pulmonary disease was 8.1 percentage points higher among bisexual adults than among gay/lesbian adults and 7.0 percentage points higher than among straight adults. These differences remained in the multivariate analyses. Additional differences were found in the sex-stratified analyses. No significant differences were found in MCC by sexual orientation.

**Conclusion:**

After age adjustment and controlling for sociodemographic characteristics, only a few significant health disparities for diagnosed chronic conditions were found by sexual orientation, and none for MCC. However, for conditions where differences were found, magnitudes were relatively large. Further examination of these differences among gay/lesbian and bisexual adults could yield a better understanding of why these disparities exist.

## Introduction

In recent years, the number of studies that examine health disparities in the United States by sexual orientation increased. Collectively these studies examined various lesbian, gay, and bisexual (LGB), or sexual minority health-related topics, including health risk behaviors (1–6), health care access (4–6), and health care service utilization (2,5–8). However, additional research is not only warranted but needed in numerous areas, one of which is the examination of chronic health conditions among LGB populations (9).

Although chronic conditions among LGB populations are not completely unexamined, only a few studies explored this topic using nationally representative samples (10,11). Most research on chronic conditions by sexual orientation has been state-level population-based studies generalizable only to the US states in which they were carried out, such as Washington (2,3,5), North Carolina (4), Massachusetts (6,12), or California (13). Conditions examined in these studies include arthritis (2,5), asthma (3,5,6,11), cancer (12), cardiovascular disease (2,4,6,10,13), diabetes (2,5,6), high cholesterol (2), and hypertension (2,5), with results varying depending on the sample used. These studies did not examine whether the conditions co-occurred as multiple (≥2) chronic conditions (MCC). Determining the prevalence of MCC among sexual minorities is important: one in 4 US adults has MCC (14,15), and recent national initiatives have addressed MCC (16–18). In addition, the care and management of MCC is more costly and complex than that of a single condition (19). 

The objective of this study was to use a descriptive epidemiological approach to examine 10 diagnosed chronic conditions and MCC by sexual orientation using a large, nationally representative sample of the civilian, noninstitutionalized US adult population. All chronic conditions selected for this study were included in previous research on MCC (14,15,17); some conditions (ie, hepatitis, chronic obstructive pulmonary disease [COPD], weak/failing kidneys) may not have previously been examined by sexual orientation using nationally representative data.

## Methods

### Data source

Data from the 2013 National Health Interview Survey (NHIS) were used. The NHIS is a multipurpose health survey of US households conducted continuously throughout the year and released annually. It serves as a primary source of health data on the civilian, noninstitutionalized US population. Data collection for the NHIS is conducted using computer-assisted personal interviewing; when necessary, interviewers may complete portions of the interview by telephone (20,21). Data used in this study came from 2 of 4 NHIS modules: the Family Core and the Sample Adult Core. The other 2 modules (Household Composition and Sample Child Core) were not used because they either had no variables needed for our analyses or they had data only on children or adolescents (<18 y). The Family Core questionnaire collects information on all family members. In addition, 1 sample adult (≥18 y) from each participating family is randomly selected to answer the Sample Adult Core questionnaire. All sample adult respondents answer for themselves unless mentally or physically unable to do so, in which case a knowledgeable family member serves as a proxy respondent (20,21).

Data analyzed in this study were collected from 34,557 sample adults and are representative of the US adult civilian, noninstitutionalized population; 571 identified as gay/lesbian, and 233 identified as bisexual. The conditional response rate (number of completed sample adult interviews divided by the total number of eligible sample adults) was 81.7%; the final response rate (product of the conditional response rate and the final family response rate) was 61.2% (20,21). 

### Measures

The chronic conditions selected for this study originally stemmed from the HHS Initiative on Multiple Chronic Conditions Strategic Framework (16,18). This framework has 4 overarching goals, one of which is to “facilitate research to fill knowledge gaps about . . . individuals with multiple chronic conditions” (18). All chronic conditions examined in this study were diagnosed conditions determined by survey questions asking adults whether they had ever been told by a doctor or health professional that they had a specific condition. Exceptions to this lifetime diagnosis measure were current asthma (measured as currently being diagnosed with asthma), COPD (measured by ever having been diagnosed with emphysema or COPD or having been diagnosed with chronic bronchitis in the past 12 months), and weak/failing kidneys (having been diagnosed in the past 12 months). Indicator variables for the 10 chronic conditions were the following: cancer (including nonmelanoma skin cancers), hypertension, coronary heart disease (CHD), stroke, COPD, current asthma, diabetes, arthritis (including rheumatoid arthritis, gout, lupus, or fibromyalgia), hepatitis, and weak/failing kidneys. These indicator variables were summed to create a categorical measure for number of conditions present: 0, 1, 2 or 3, and 4 or more (14). Although research on MCC suggests including 20 chronic conditions when measuring number of chronic conditions, data are collected in the NHIS for only the 10 aforementioned conditions (14,17).

Sexual orientation was determined by a survey question that asked, “Which of the following best represents how you think of yourself?” For men, responses were gay; straight, that is not gay; bisexual; something else; and “I don’t know the answer.” For women, response categories were lesbian or gay; straight, that is not lesbian or gay; bisexual; something else; and “I don’t know the answer.” Methodological considerations for this survey question are detailed elsewhere (20).

Measures for sociodemographic characteristics used in this study were sex, age group, race/ethnicity, education level, total annual combined family income (taken from the NHIS imputed income data) (22), marital status, place of residence, health insurance status, and employment status.

### Statistical analyses

Although crude estimates would show the actual burden of chronic conditions by sexual orientation among US adults, estimates from the NHIS show that chronic conditions and MCC are strongly and positively associated with age (15), whereas self-identifying as gay, lesbian, or bisexual is negatively associated with age (7). To help negate any potential influence of age on the comparison of the prevalence of chronic conditions by sexual orientation, the estimates were age-adjusted. Age-adjusted prevalence of selected chronic conditions and number of chronic conditions were estimated for the total adult population and separately for gay/lesbian, straight, and bisexual adults (with those 0.2% of adults who identified as “something else” and 0.4% of adults who responded “I don’t know the answer” dropped from the denominator). Sex-stratified estimates were also generated. Age-adjustment was performed using the 2000 US Census–projected population using the following 3 age groups: 18–44 years, 45–64 years, and ≥65 years (23). Two-tailed significance tests were used to determine significant differences in the prevalence of chronic conditions among gay/lesbian, straight, and bisexual adults. All prevalence differences discussed were considered significant at the *P* < .05 level and were not adjusted for multiple comparisons.

In addition to these estimates, multivariate logistic regression analyses were conducted. An indicator of each of the selected diagnosed chronic conditions was regressed on sexual orientation (using “straight” as the reference category) and sociodemographic characteristics. Models were estimated for all US adults and for men and women separately (results not adjusted for multiple comparisons). Because our analyses showed no difference in the prevalence of MCC by sexual orientation, no multivariate analyses were conducted for this outcome.

Analyses were weighted using NHIS survey weights to allow for generalization to the US adult civilian, noninstitutionalized population. Survey design variables were incorporated to account for covariance resulting from the complex cluster sample design of the NHIS using SUDAAN version 10.0.1 (RTI International). Because of the relatively small estimated size of the sexual minority population (2.3% of adults per NHIS) (7) and the resulting small sample sizes in national surveys, the use of national surveys may yield estimates that require cautious interpretation. Thus, prevalence estimates are reported according to the current standards used by the National Center for Health Statistics, where estimates with a relative standard error (RSE) less than 30.0% are considered reliable. RSEs are calculated as the standard error of an estimate divided by the estimate itself, multiplied by 100.

## Results

Approximately 1.6% of adults identified as gay or lesbian and 0.7% as bisexual; 48.2% were men; and 47.1% were aged 18 to 44 (Table 1). Hypertension and arthritis were the 2 most prevalent chronic conditions among all sexual-orientation groups, all adults, and among men and women separately (Table 2), although confidence intervals for prevalence rates for certain conditions overlap and warrant caution when determining rank order.

**Table 1:** Percentage Distribution of Selected Sociodemographic Characteristics of US Adults, National Health Interview Survey, 2013^a^

Characteristic	%^b^ (95% CI)
**Sexual orientation**	
Gay/lesbian	1.6 (1.4–1.8)
Straight	97.2 (96.9–97.4)
Bisexual	0.7 (0.6–0.8)
Something else	0.2 (0.1–0.3)
I don’t know the answer	0.4 (0.3–0.5)
**Sex**	
Male	48.2 (47.5–48.9)
Female	51.8 (51.1–52.5)
**Age, y**	
18–44	47.1 (46.2–47.9)
45–64	34.7 (33.9–35.4)
≥65	18.3 (17.7–18.9)
**Race/ethnicity**	
Hispanic	15.0 (14.4–15.7)
Non-Hispanic white	66.1 (65.2–66.9)
Non-Hispanic black	11.5 (11.0–12.1)
Non-Hispanic Asian	5.4 (5.0–5.8)
Non-Hispanic other^c^	2.0 (1.7–2.2)
**Education level**	
Less than high school	13.8 (13.2–14.3)
High school diploma/GED	26.1 (25.4–26.8)
Some college	30.9 (30.2–31.6)
Bachelor’s degree or higher	29.2 (28.4–30.1)
**Total annual combined family income, $**	
≤24,999	22.2 (21.5–23.0)
25,000–49,999	23.9 (23.2–24.6)
50,000–74,999	18.6 (18.0–19.2)
≥75,000	35.3 (34.4–36.3)
**Married/cohabitating**	
No	40.0 (39.2–40.8)
Yes	60.0 (59.2–60.8)
**Place of residence**	
Not in a metropolitan statistical area	15.2 (13.9–16.6)
Small metropolitan statistical area	30.9 (29.5–32.4)
Large metropolitan statistical area	53.9 (52.6–55.2)
**Health insurance status**	
Private	62.1 (61.3–62.9)
Public	17.9 (17.3–18.5)
Other	3.4 (3.1–3.6)
Uninsured	16.6 (16.0–17.3)
**Employed in the past 12 months**	
No	32.9 (32.2–33.6)
Yes	67.1 (66.4–67.8)
**Age by sexual orientation, y**	
Gay/lesbian	
18–44	54.4 (48.8–59.8)
45–64	38.1 (32.9–43.6)
≥65	7.5 (5.2–10.7)
Straight	
18–44	46.8 (46.0–47.7)
45–64	34.7 (33.9–35.4)
≥65	18.5 (17.9–19.1)
Bisexual	
18–44	76.1 (68.7–82.3)
45–64	19.1 (13.5–26.2)
≥65	4.8 (2.6–8.6)

Abbreviations: CI, confidence interval; GED, general educational development.

a Estimates generated using the 2013 National Health Interview Survey Sample Adult data file (n = 34,557). Estimates are crude estimates.

b Percentages may not add to 100 because of rounding.

c Includes adults who identified with multiple racial groups.

**Table 2:** Age-Adjusted Prevalence of Selected Diagnosed Chronic Conditions Among Adults, by Sexual Orientation and Sex, United States, 2013^a^

Condition^b^	% (95% CI)			
	Total	Gay/Lesbian	Straight	Bisexual
**All adults**				
Hypertension	27.8 (27.2–28.3)	26.2 (21.7–31.2)	27.8 (27.2–28.4)	24.1 (17.2–32.7)
Arthritis	21.2 (20.7–21.8)	26.7 (22.1–31.7)^c^	21.1 (20.6–21.7)^d^	22.5 (15.7–31.0)
Diabetes	8.7 (8.3–9.0)	6.7 (4.6–9.6)	8.7 (8.4–9.1)	9.7 (5.4–17.0)
Cancer	7.9 (7.5–8.2)	9.6 (7.0–13.0)^e^	7.8 (7.5–8.2)	4.9 (2.5–9.6)^d^^,^ f
Current asthma	6.9 (6.5–7.3)	8.6 (6.4–11.6)	6.8 (6.4–7.2)	11.7 (7.3–18.2)
COPD	5.7 (5.4–6.0)	4.6 (2.9–7.1)^e^	5.7 (5.3–6.0)^e^	12.7 (7.7–20.2)^c^^,^ d
CHD	4.4 (4.2–4.6)	2.9 (1.8–4.9)	4.4 (4.2–4.6)	4.4 (1.8–10.1)^f^
Hepatitis	2.6 (2.4–2.8)	5.5 (3.7–8.2)^c^	2.6 (2.4–2.8)^d^	11.5 (6.6–19.1)
Stroke	2.5 (2.4–2.8)	1.6 (0.8–3.5)^f^	2.6 (2.4–2.8)	— g
Weak/failing kidneys	1.7 (1.5–1.9)	2.1 (0.9–4.9)^f^	1.7 (1.5–1.9)	— g
**Men**				
Hypertension	28.9 (28.1–29.8)	25.8 (20.2–32.4)	29.0 (28.2–29.9)	29.2 (19.6–41.1)
Arthritis	18.0 (17.3–18.7)	17.8 (13.0–23.9)	17.9 (17.2–18.7)	27.0 (18.6–37.4)
Diabetes	9.2 (8.6–9.7)	8.6 (5.4–13.6)	9.2 (8.6–9.7)	13.5 (6.8–25.1)^f^
Cancer	7.4 (6.9–7.9)	13.2 (9.3–18.4)^c^^,^ e	7.3 (6.8–7.8)^d^	5.8 (2.2–14.2)^d^^,^ f
Current asthma	5.1 (4.7–5.6)	7.8 (5.1–11.9)	5.0 (4.6–5.5)	9.0 (3.4–21.2)^f^
COPD	5.0 (4.6–5.4)	3.3 (1.9–5.8)	4.9 (4.5–5.4)	11.0 (4.8–23.1)^f^
CHD	5.8 (5.4–6.3)	4.2 (2.4–7.2)	5.8 (5.4–6.3)	7.9 (3.2–18.5)^f^
Hepatitis	2.7 (2.4–3.1)	7.4 (4.6–11.7)^c^	2.6 (2.3–3.0)^d^	16.7 (9.6–27.4)
Stroke	2.6 (2.4–3.0)	— g	2.7 (2.4–3.0)	— g
Weak/failing kidneys	1.6 (1.4–1.9)	1.7 (0.8–3.9)^f^	1.6 (1.4–1.8)	— g
**Women**				
Hypertension	26.6 (25.9–27.3)	26.4 (20.2–33.9)	26.6 (25.9–27.4)	17.7 (9.8–29.7)
Arthritis	24.2 (23.4–24.9)	36.3 (29.5–43.7)^c^^,^ e	24.0 (23.2–24.8)^d^	15.8 (8.7–27.0)^d^
Diabetes	8.2 (7.8–8.7)	4.5 (2.5–8.0)^c^	8.3 (7.8–8.8)^d^	— g
Cancer	8.4 (7.9–8.9)	5.7 (3.3–9.7)	8.4 (8.0–9.0)^e^	2.5 (1.2–5.3)^c^^,^ f
Current asthma	8.6 (8.0–9.1)	9.5 (6.2–14.4)	8.5 (7.9–9.0)	12.4 (7.3–20.4)
COPD	6.4 (6.0–6.9)	6.0 (3.2–11.0)^f^	6.4 (5.9–6.8)	13.6 (6.9–25.2)^f^
CHD	3.2 (2.9–3.5)	— g	3.2 (2.9–3.5)	— g
Hepatitis	2.5 (2.3–2.8)	3.4 (1.5–7.4)^f^	2.5 (2.3–2.8)	— g
Stroke	2.5 (2.2–2.7)	2.4 (0.9–6.2)^f^	2.4 (2.2–2.7)	— g
Weak/failing kidneys	1.8 (1.6–2.1)	— g	1.8 (1.6–2.1)	— g

Abbreviations: CHD, coronary heart disease; CI, confidence interval; COPD, chronic obstructive pulmonary disease.

a Estimates generated using the 2013 National Health Interview Survey Sample Adult data file (n = 34,557).

b Chronic conditions are listed in descending order of prevalence for all adults (total). CIs for certain conditions may overlap and warrant caution in determining rank order. All conditions were measured as ever having been told by a doctor/health professional, with the exception of current asthma (currently being diagnosed with asthma), COPD (ever having been diagnosed with emphysema or COPD, or having been diagnosed with chronic bronchitis in the past 12 months), and weak/failing kidneys (having been diagnosed in the past 12 months). Age-adjustment was to the 2000 US Census projected population using 3 age groups: 18–44, 45–64, and ≥65 years.

c Prevalence of this condition was significantly different (2-tailed; *P* < .05) from those who identified as straight.

d Prevalence of this condition was significantly different (2-tailed; *P* < .05) from those who identified as gay/lesbian.

e Prevalence of this condition was significantly different (2-tailed; *P* < .05) from those who identified as bisexual.

f Estimates that have a relative standard error (RSE) >30.0% and ≤50.0% and should be interpreted with caution.

g Estimates have an RSE >50.0%, are considered unreliable, and are not reported.

Some differences in the age-adjusted prevalence of chronic conditions by sexual orientation were found. In only 2 instances was prevalence higher among straight adults than among sexual minority adults: the prevalence of diabetes was 3.8 percentage points higher among straight women than among gay/lesbian women (8.3% vs 4.5%), and the prevalence of cancer was 5.9 percentage points higher among straight women than among bisexual women (8.4% vs 2.5%).

COPD was more than twice as prevalent among bisexual adults (12.7%) than among gay/lesbian adults (4.6%) or straight adults (5.7%). However, other significant differences showed that gay/lesbian adults had a higher prevalence of some conditions than other sexual orientation groups had — among adults overall and stratified by sex. For example, the prevalence of arthritis was 5.6 percentage points higher among gay/lesbian adults than among straight adults (26.7% vs 21.1%), and the prevalence of hepatitis was 2.9 percentage points higher among gay/lesbian adults than among straight adults (5.5% vs 2.6%). The sex-stratified analyses showed this pattern also for arthritis among women (36.3% for gay/lesbian women vs 24.0% for straight women; a 12.3 percentage-point difference). This same pattern was also found for cancer and hepatitis among men. Cancer was 5.9 percentage points more prevalent among gay men (13.2%) than among straight men (7.3%); hepatitis was 4.8 percentage points more prevalent among gay men (7.4%) than among straight men (2.6%). Cancer was 4.7 percentage points more prevalent among gay/lesbian adults than among bisexual adults (9.6% vs. 4.9%), and it was more than twice as prevalent among gay men as among bisexual men (13.2% vs 5.8%). Arthritis was more than twice as prevalent among gay/lesbian women as among bisexual women (36.3% vs 15.8%).

After controlling for sociodemographic factors, only some significant differences in diagnosed chronic conditions by sexual orientation remained (Table 3). Gay/lesbian adults had moderately higher odds of arthritis compared with straight adults (adjusted odds ratio [AOR] = 1.52); the same was found for women (AOR = 2.14). Gay men had higher odds of cancer compared with straight men (AOR = 2.09). Higher odds of hepatitis were found for gay/lesbian adults (AOR = 2.16) compared with straight adults and for gay men compared with straight men (AOR = 2.86). When models were run using the bisexual group as the reference category (data not shown in tables), gay/lesbian adults (AOR = 0.42; 95% confidence interval [CI], 0.20–0.90) and straight adults (AOR = 0.50; 95% CI, 0.27–0.91) adults had lower odds of COPD than bisexual adults.

**Table 3:** Logistic Regression of Sexual Orientation and Sociodemographic Characteristics on Selected Diagnosed Chronic Conditions Among Adults, United States, 2013^a^

Condition^b^	All Adults^c^		Men^d^		Women^d^	
	AOR (95% CI)	*P* Value	AOR (95% CI)	*P* Value	AOR (95% CI)	*P* Value
**Hypertension**						
Straight	1 [Reference]	—	1 [Reference]	—	1 [Reference]	—
Gay/lesbian	0.97 (0.73–1.30)	.84	0.94 (0.65–1.37)	.76	1.08 (0.70–1.66)	.73
Bisexual	0.78 (0.49–1.23)	.29	1.03 (0.55–1.92)	.93	0.66 (0.34–1.27)	.21
**Arthritis**						
Straight	1 [Reference]	—	1 [Reference]	—	1 [Reference]	—
Gay/lesbian	1.52 (1.14–2.05)	.005	1.07 (0.68–1.68)	.78	2.14 (1.45–3.17)	<.001
Bisexual	1.05 (0.65–1.71)	.83	2.12 (1.11–4.03)	.02	0.74 (0.38–1.44)	.37
**Diabetes**						
Straight	1 [Reference]	—	1 [Reference]	—	1 [Reference]	—
Gay/lesbian	0.95 (0.62–1.46)	.83	1.11 (0.65–1.92)	.70	0.85 (0.42–1.72)	.65
Bisexual	0.88 (0.46–1.67)	.69	1.70 (0.70–4.13)	.24	0.46 (0.18–1.22)	.12
**Cancer**						
Straight	1 [Reference]	—	1 [Reference]	—	1 [Reference]	—
Gay/lesbian	1.27 (0.86–1.87)	.24	2.09 (1.26–3.46)	.004	0.76 (0.40–1.42)	.39
Bisexual	0.86 (0.44–1.70)	.67	0.72 (0.25–2.13)	.56	0.80 (0.35–1.83)	.59
**Current asthma**						
Straight	1 [Reference]	—	1 [Reference]	—	1 [Reference]	—
Gay/lesbian	1.32 (0.94–1.85)	.12	1.60 (0.96–2.65)	.07	1.11 (0.70–1.76)	.66
Bisexual	1.58 (0.95–2.63)	.08	1.86 (0.62–5.58)	.27	1.53 (0.87–2.70)	.14
**COPD**						
Straight	1 [Reference]	—	1 [Reference]	—	1 [Reference]	—
Gay/lesbian	0.87 (0.55–1.38)	.55	0.82 (0.41–1.64)	.57	0.91 (0.48–1.72)	.77
Bisexual	1.97 (1.07–3.60)	.03	2.44 (0.93–6.41)	.07	1.72 (0.82–3.60)	.15
**CHD**						
Straight	1 [Reference]	—	1 [Reference]	—	1 [Reference]	—
Gay/lesbian	0.91 (0.52–1.59)	.75	1.17 (0.61–2.24)	.63	0.54 (0.20–1.45)	.22
Bisexual	1.17 (0.44–3.10)	.76	1.71 (0.55–5.30)	.36	0.46 (0.06–3.65)	.46
**Hepatitis**						
Straight	1 [Reference]	—	1 [Reference]	—	1 [Reference]	—
Gay/lesbian	2.16 (1.40–3.33)	<.001	2.86 (1.74–4.72)	<.001	1.48 (0.60–3.66)	.40
Bisexual	2.38 (1.20–4.75)	.01	5.24 (2.08–13.21)	<.001	0.86 (0.29–2.57)	.78
**Stroke**						
Straight	1 [Reference]	—	1 [Reference]	—	1 [Reference]	—
Gay/lesbian	0.66 (0.31–1.44)	.30	0.37 (0.13–1.09)	.07	1.17 (0.42–3.22)	.77
Bisexual	1.28 (0.47–3.47)	.63	0.27 (0.05–1.46)	.13	1.85 (0.62–5.54)	.27
**Weak/failing kidneys**						
Straight	1 [Reference]	—	1 [Reference]	—	1 [Reference]	—
Gay/lesbian	1.18 (0.56–2.48)	.66	1.39 (0.59–3.25)	.45	0.94 (0.26–3.46)	.93
Bisexual	1.29 (0.40–4.14)	.67	3.41 (0.72–16.22)	.12	0.47 (0.10–2.17)	.34

Abbreviations: AOR, adjusted odds ratio; CHD, coronary heart disease; CI, confidence interval; COPD, chronic obstructive pulmonary disease; GED, general educational development; MSA, metropolitan statistical area.

a Estimates generated using the 2013 National Health Interview Survey Sample Adult data file (n = 34,557).

b All conditions were measured as ever having been told by a doctor/health professional, with the exception of current asthma (currently being diagnosed with asthma), COPD (ever having been diagnosed with emphysema or COPD, or having been diagnosed with chronic bronchitis in the past 12 months) and weak/failing kidneys (having been diagnosed in the past 12 months).

c Covariates included in models (but not shown in table) are the following: sex (1 = male), age group (18–44 y, 45–64 y, ≥65 y), race/ethnicity (Hispanic, non-Hispanic white, non-Hispanic black, non-Hispanic Asian, non-Hispanic other race), educational level (less than high school, high school diploma/GED, some college, bachelor’s degree or higher), total annual combined family income (<$24,999, $25,000–$49,999, $50,000–$74,999, ≥$75,000), married/cohabitating (1 = yes), place of residence (not in an MSA, small MSA, large MSA), health insurance status (private, public, other, uninsured), and employed in the past 12 months (1 = yes).

d Covariates included in models (but not shown in table) are the following: age group (18–44 years, 45–64 years, ≥65 years), race/ethnicity (Hispanic, non-Hispanic white, non-Hispanic black, non-Hispanic Asian, non-Hispanic other race), educational level (less than high school, high school diploma/GED, some college, bachelor’s degree or higher), total annual combined family income (<$24,999, $25,000–$49,999, $50,000–$74,999, ≥$75,000), married/cohabitating (1 = yes), place of residence (not in a MSA, small MSA, large MSA), health insurance status (private, public, other, uninsured), and employed in the past 12 months (1 = yes).

The multivariate analysis showed suppression effects in some instances: significant differences not found in the bivariate analysis were found in the multivariate analysis. In the multivariate analysis (Table 3), bisexual adults had higher odds of hepatitis compared with straight adults (AOR = 2.38; 95% CI, 1.20–4.75). Bisexual men had higher odds of arthritis (AOR = 2.12; 95% CI, 1.11–4.03) and hepatitis (AOR = 5.24; 95% CI, 2.08–13.21) compared with straight men. 

An examination of the number of chronic conditions found no significant differences among gay/lesbian, straight, and bisexual adults having MCC (either 2 or 3 conditions or ≥4 conditions) (Table 4). Furthermore, no differences were found in the percentages of adults who reported a single condition or no chronic conditions. Thus, accounting for age effects resulted in no significant differences in MCC by sexual orientation.

**Table 4:** Age-Adjusted Prevalence of Selected Diagnosed Multiple Chronic Conditions Among Adults, by Sexual Orientation and Sex, United States, 2013^a^

Condition^b^	% (95% CI)			
	Total	Gay/Lesbian	Straight	Bisexual
**All adults**				
0 conditions	52.7 (52.0–53.3)	47.8 (42.8–52.7)	52.8 (52.2–53.5)	49.4 (40.9–58.0)
1 condition	23.5 (22.9–24.1)	25.8 (21.4–30.9)	23.4 (22.8–24.0)	27.5 (20.2–36.2)
2 or 3 conditions	19.3 (18.8–19.7)	23.0 (18.8–27.8)	19.2 (18.7–19.7)	15.7 (10.4–23.2)
≥4 conditions	4.6 (4.3–4.9)	3.5 (2.2–5.4)	4.6 (4.3–4.9)	7.4 (3.6–14.5)^c^
**Men**				
0 conditions	54.1 (53.2–55.0)	48.7 (42.5–55.1)	54.2 (53.3–55.2)	45.6 (36.0–55.6)
1 condition	22.9 (22.0–23.8)	26.1 (20.5–32.5)	22.9 (22.0–23.8)	29.7 (18.5–44.0)
2 or 3 conditions	18.5 (17.7–19.2)	22.1 (16.8–28.4)	18.4 (17.6–19.1)	15.3 (8.6–25.8)
≥4 conditions	4.5 (4.1–4.9)	3.1 (1.7–5.8)^c^	4.5 (4.1–4.9)	9.4 (3.9–21.0)^c^
**Women**				
0 conditions	51.3 (50.4–52.2)	46.6 (38.7–54.6)	51.4 (50.5–52.4)	56.3 (43.9–67.9)
1 condition	24.0 (23.2–24.8)	25.8 (19.6–33.1)	23.9 (23.1–24.7)	24.9 (17.4–34.4)
2 or 3 conditions	20.0 (19.3–20.7)	23.9 (18.1–31.0)	20.0 (19.3–20.7)	14.1 (7.7–24.7)^c^
≥4 conditions	4.7 (4.3–5.5)	3.8 (2.0–7.2)^c^	4.7 (4.3–5.0)	— d

Abbreviations: CI, confidence interval; COPD, chronic obstructive pulmonary disease; CHD, coronary heart disease.

a Estimates were generated using the 2013 National Health Interview Survey Sample Adult data file (n = 34,557).

b Ten chronic conditions were included in the study: hypertension, arthritis, diabetes, cancer, current asthma, COPD, CHD, hepatitis, stroke, and weak/failing kidneys. All conditions were measured as ever having been told by a doctor/health professional, with the exception of current asthma (currently being diagnosed with asthma), COPD (ever having been diagnosed with emphysema or COPD, or having been diagnosed with chronic bronchitis in the past 12 months) and weak/failing kidneys (having been diagnosed in the past 12 months). Age-adjustment was to the 2000 US Census projected population using 3 age groups: 18–44 years, 45–64 years, and ≥65 years.

c Estimates have a relative standard error (RSE) >30.0% and ≤50.0% and should be interpreted with caution.

d Estimates have a RSE>50.0%, are considered unreliable, and are not reported.

## Discussion

Age-adjusted estimates showed that among US adults, by sex and by sexual-orientation groups, hypertension was the most prevalent chronic condition, followed by arthritis. For certain conditions, significant differences among gay/lesbian, straight, and bisexual adults were found. The prevalence of cancer was higher among gay/lesbian adults than among bisexual adults (although this difference was not significant in the multivariate analyses), and hepatitis and arthritis were more likely to be found among gay/lesbian adults than among straight adults. The findings on arthritis are consistent with those of 1 study that found a higher likelihood of arthritis among gay/lesbian women compared with straight women (13) but are inconsistent with those of another study, which did not find this difference (2).

Bisexual adults had a higher likelihood of COPD than either gay/lesbian adults or straight adults. Additional differences by sexual-orientation group were found in the sex-stratified analyses. These initial stratified analyses found only 2 instances in which estimates were significantly higher among straight adults than among sexual minority groups: the prevalence of diabetes was higher among straight women than among gay/lesbian women, and the prevalence of cancer was higher among straight women than among bisexual women. However, after controlling for sociodemographic characteristics, these 2 differences were not found; this absence of significant differences for diabetes is consistent with previous research (2,4,6,24). Although previous studies found disparities in cardiovascular disease and symptoms (2,4,6,13) and asthma (6,24) by sexual orientation, others did not (4,6,24). Our study found no differences in CHD or asthma prevalence by sexual orientation among all adults or among men and women separately. In addition, no differences in the prevalence of MCC were found by sexual orientation among all adults or among men and women separately.

Although the number of significant differences found was not large, the magnitude of some of these differences was large, and remained even after controlling for sociodemographic variables. For example, COPD was more than 2 times as prevalent among bisexual adults as it was among gay/lesbian or straight adults. The significant differences were also large in magnitude in the sex-stratified analyses; for example, gay/lesbian women were twice as likely to have arthritis as bisexual women. Thus, although the number of differences in diagnosed chronic conditions by sexual orientation may be small, the size of these disparities is notable and warrants further examination.

This study has several limitations. The NHIS captures data only on conditions diagnosed by a physician or other health professional; underestimations may result from not including conditions that are not diagnosed. Underestimation may be further compounded in this study because sexual minority groups may have less access to health care (7) and subsequently may be more likely to have undiagnosed chronic conditions. In addition, because NHIS data are mainly self-reported, underreporting may result from a lack of respondent recall. Mental health conditions (ie, depression, substance use disorders), which in recent years are more frequently considered along with physical chronic diseases when studying comorbidities, were not included in this study. Furthermore, only 10 chronic conditions were examined. Although this number is in line with the operational definition of MCC outlined by Goodman and colleagues (17), it only begins to cover the range of chronic conditions among adults in the United States, and it does not include several conditions for which prevalence differs by sexual orientation (eg, HIV, obesity) (7). From a measurement perspective, potential limitations were the small sample sizes of sexual minority groups, which in some instances resulted in unreliable prevalence estimates and wide confidence intervals, and the omission of ascertaining other sexual and gender-minority groups, for example, those identifying as transgender (20). Multiple comparisons were made in this study, which increased the possibility of committing type I errors. Finally, although the NHIS adjusts its sampling weights for nonresponse, a final response rate of 61.2% for the NHIS Sample Adult Core may also be a limitation.

Nevertheless, this descriptive study may be the first nationally representative examination focused solely on diagnosed chronic conditions and MCC among sexual minority groups, and for some conditions (eg, hepatitis, COPD, weak/failing kidneys), our prevalence estimates may be the first national estimates. Where disparities were found, future research could investigate underlying causes. For example, health and sociodemographic characteristics, such as health-risk behaviors, health care access, care management, or use of health care services, may explain the large differences by sexual orientation in the prevalence of COPD, cancer, and arthritis. Furthermore, numerous additional chronic conditions, such as liver disease and other types of cardiovascular disease, including angina pectoris and myocardial infarction, are yet to be explored for differences by sexual orientation using NHIS data. Only by further researching chronic conditions among sexual minority populations can a better understanding of disparities (or lack of disparities, as for MCC) be achieved.
