# Erratum, Vol. 12, November 5 Release

**DOI:** 10.5888/pcd12.150292e

**Published:** 2015-12-31

In the article “Selected Diagnosed Chronic Conditions by Sexual Orientation: A National Study of US Adults, 2013,” because of a technical error, the footnotes for Table 4 were incorrect. The footnotes were corrected on November 30, 2015, and appear online at http://www.cdc.gov/pcd/issues/2015/15_0292.htm. We regret any confusion this error may have caused.

